# Increased sensitivity of an infrared motility assay for nematicide discovery

**DOI:** 10.17912/micropub.biology.000500

**Published:** 2021-11-30

**Authors:** Franco Vairoletti, Antonela Baron, Cecilia Saiz, Graciela Mahler, Gustavo Salinas

**Affiliations:** 1 Laboratorio de Química Farmacéutica, Departamento de Química Orgánica, Facultad de Química, Universidad de la República, Montevideo, Uruguay; 2 Graduate Program in Chemistry, Facultad de Química, Universidad de la República, Uruguay.; 3 Phylumtech S.A. Belgrano 758, Sunchales, Santa Fe, Argentina.; 4 Laboratorio de Química Farmacéutica, Departamento de Química Orgánica, Facultad de Química, Universidad de la República, Uruguay; 5 Laboratorio de Química Farmacéutica, Departamento de Química Orgánica, Facultad de Química, Universidad de la República, Montevideo, Uruguay; 6 Worm Biology Laboratory, Institut Pasteur de Montevideo, Mataojo 2020, Montevideo 11400, Uruguay; 7 Departamento de Biociencias, Facultad de Química, Universidad de la República, Avda. Gral Flores 2124, Montevideo 11300, Uruguay

## Abstract

Parasitic nematodes constitute a health problem for humans, livestock and crops, and cause huge economic losses to developing-country economies. Due to the spread of nematicide resistance, there is an urgent need for new drugs. *C. elegans* is now recognized as a cost-effective alternative for the screening of compound libraries with potential nematicidal activity, as parasitic organisms are hard to maintain under laboratory conditions. Here we describe an adaptation of a previously reported high throughput (HTP) infrared-based motility assay that leads to increased sensitivity. The modified assay uses L1 instead of L4 stage worms and matches the sensitivity reported by Burns et al. (2015) for the anthelmintic benzamides Wact11 and Wact11p. In addition, this method presents practical advantages over Burns et al. (2015) and other image-based protocols and provides a robust assay with a fast and simple readout ideal for HTP drug discovery.

**Figure 1. L1-adapted infrared motility assay provides enhanced sensitivity for nematicide discovery:**
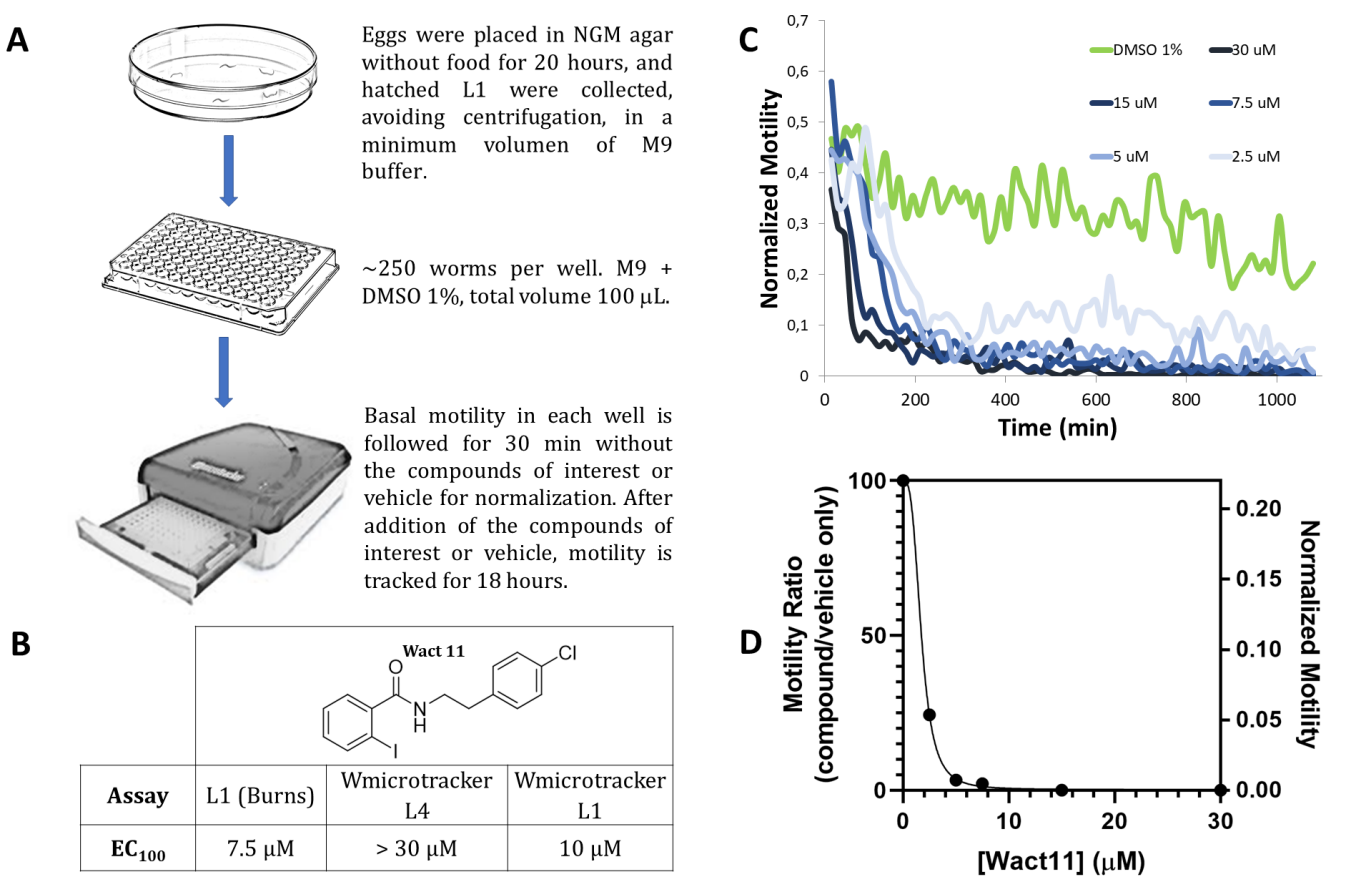
**A.**Adapted protocol described using L1 larvae synchronized in liquid medium. **B.** Comparison of EC_100_ values obtained for Wact11 by Burns *et al.* (L1) and our L4 and adapted L1 assay*.* **C.** Kinetic data from a representative Wact11 dose-response experiment, Curves for vehicle (DMSO 1%) and different compound concentrations in vehicle are shown. **D.** Dose-response curve for Wact11 at endpoint in our adapted L1 assay. The motility ratio is defined as compound normalized motility in vehicle/vehicle motility.

## Description

Nematodes are a diverse phylum that includes numerous lineages that parasitize humans, animals, and terrestrial plants, being a health threat to humans, livestock and crops that heavily impacts food production and economies, particularly in developing countries. The main control strategy for nematode-caused infections relies on chemotherapy, but there are few compound families available and their efficacy is seriously compromised due to resistance dissemination, chiefly in crops and livestock. This demands an urgent need for new nematicides to be used in field applications. (Caffrey *et al.* 2012)

One of the main obstacles to designing and discovering new nematicidal agents is the lack of efficient screening assays to assess the activity of compound libraries. The free-living nematode *Caenorhabditis elegans*, a widely used model in biology, is currently recognized as a cost-effective alternative HTP of either synthetic or natural product libraries (Salinas and Risi 2018).

Within the framework of a nematicide discovery project, we initially took advantage of a previously described infrared-based motility method using *C. elegans* L4 larvae and the WMicrotracker device, (Gunderson *et al.* 2020) which automatically monitors the motility of the worms as a vitality proxy. The assay allowed to screen both natural product and synthetic libraries and was validated with commercial nematicides. (Risi *et al.* 2019). However, when Wact11 and Wact11p, two members of the nematicidal benzamide family, were assayed through this method, we found a notable discrepancy between the activity values previously reported for these compounds and those obtained with WMicrotracker, being the apparent potency much lower in the latter. Burns *et al.* report a LD_100_ of 7.5 µM for both Wact11 and Wact11p. In the infrared-based assay, a total death/paralysis phenotype was not observed even at 30 µM, the highest concentration tested (Figure, panel B). Furthermore, when applied to an in-house synthesized chemolibrary, the original L4 assay precluded us from extracting information about the structure-activity relationships of derivatives, as all of them were indistinguishable from the vehicle control. This compromised the utility of the assay to guide the design of new derivatives.

To understand the difference and overcome this obstacle, we examined in detail the protocol developed by Burns *et al.* in comparison with the WMicrotracker assay. The main differences are the larval stage employed, the assay time, the monitoring method and the presence of *E. coli* as a food source during incubation with tested compounds.

We reasoned that the crucial difference between both assays was the worm larval stage. L1 larvae could be inherently more sensitive to xenobiotic aggression than L4 due to cuticle permeability (Hartman *et al.* 2021) or to metabolic differences. Thus, to increase the sensitivity of the infrared-motility assay we used a direct adaptation of the original L4 protocol using L1 larvae synchronized in liquid medium, keeping all other assay parameters constant. However, this change did not allow reasonable basal motility to be detected, even increasing the number of larvae per well up to 1000. We thus explored additional modifications to the original protocol, letting L1 larvae synchronize overnight without any food source in an NGM agar plate, in order to increase their basal motility. Approximately 250 of these starved L1 larvae per well in M9 + BSA 0.015% are enough to reliably detect their movement by the WMicrotracker device. Assay time, vehicle and data process were all kept unchanged. Starvation on plates seems to be crucial for movement detection of L1 larvae and allowed motility to be measured over 18 hours. For nematicide discovery this is a potential advantage, but it should be born in mind that starvation alters gene expression and metabolism, and therefore initial screens should be followed by experiments under other conditions.

When the infrared L1-adapted assay was employed to assess Wact11 activity against *C. elegans,* an EC_100_ value of 10 µM was obtained. The adapted assay resulted in a significant increase in sensitivity compared to the original L4 assay, and in close agreement with the EC_100_ value reported by Burns *et al.* (7.5 µM), The dose-response curve (Figure 1, panel D) at the endpoint showed a clear dose-dependent effect for this compound in the L1 assay. Importantly, the adapted assay features several advantages compared to Burns *et al.* protocol, such as reduced assay time (18 h vs. several days), the absence of *E. coli* during compound incubation (which could interfere by absorbing and metabolizing the compound being tested) and microscopy-independent automatized monitoring, which significantly increased throughput and efficiency. This protocol is thus a valuable alternative when testing compound libraries with nematicidal potential. Furthermore, the modifications made to the infrared motility assay could be applied to other protocols as a way to modulate sensitivity/specificity tradeoff when using *C. elegans* as a drug screening model, either in medicinal chemistry or toxicology applications.

## Methods

**Wact11 and Wact11p synthesis**

Under N_2_ atmosphere, 2-(trifluoromethyl)benzoic acid (for Wact11) or 2-iodobenzoic acid (for Wact11p) (1.1 mmol) was dissolved in dry CH_2_Cl_2_ (2 mL), then oxalyl chloride (1.6 mmol) and DMF (0.02 mL) were added dropwise. The mixture was stirred for 10 minutes at room temperature and then the solvent was removed under reduced pressure. The residue was redissolved in CH_2_Cl_2 _(2 mL) cooled into an ice bath followed by the addition of Et_3_N (1.1 mmol) and 2-(4-chlorophenyl)ethan-1-amine) (1.1 mmol). After 4 hours, the mixture was poured into water and was extracted with AcOEt. The combined organic layers were washed with HCl 5% and NaHCO_3_ 10% and dried with Na_2_SO_4_. The solvent was removed under reduced pressure and the crude was purified by silica column chromatography (CH_2_Cl_2_/Hx (3:1)) to yield Wact11 (58%) or Wact11p (76%) as a white solid. Spectroscopic data is in concordance with product structure.

***C. elegans* culture**

Wild-type *C. elegans* Bristol strain N2 and *E. coli* OP50 strain were obtained from the Caenorhabditis Genomics Center (Minneapolis, MN, USA). The worms were maintained under standard conditions at 20 °C on nematode growth media (NGM) agar plates seeded with *E. coli* OP50 as a source of food. The wild-type *C. elegans* Bristol strain N2 was cultured and maintained according to the procedures described previously (Brenner, 1974).

**L4 assay**

The L4 assay was conducted followed the protocol described in Risi *et al.* 2019.

**L1 assay**

Eggs obtained from bleaching were placed on empty magnesium complemented NGM plates (without bacteria) and let hatch for 20 hours at 20°C. L1 worms were collected in a minimum volume of M9 buffer. Approximately 250 worms were seeded in each well of a microtiter plate in 80 microliters of M9 buffer with 0.015 % BSA. Motility was tracked for 30 min. in the WMicrotracker device for normalization. After this, compounds of interest in vehicle (M9 + DMSO 1%) or vehicle alone were added in a volume of 20 microliters. Motility was tracked for additional 18 hs. Counts per well were normalized with the value obtained previously to compound or control addition.

## Reagents

Bristol N2 strain of *C. elegans*, available at CGC.

2-(trifluoromethyl)benzoic acid, 2-iodobenzoic acid and oxalyl chloride (Sigma-Aldrich). Triethylamine (Merck), 4-chlorophenyl)ethan-1-amine (Accela). Solvents were freshly distilled before the reaction.
